# n-3 Polyunsaturated Fatty Acid Supplementation Affects Oxidative Stress Marker Levels in Patients with Type II Intestinal Failure: A Randomized Double Blind Trial

**DOI:** 10.3390/antiox12081493

**Published:** 2023-07-26

**Authors:** Adriana Flores-López, Martha Guevara-Cruz, Azalia Avila-Nava, Alejandro G. González-Garay, Luis E. González-Salazar, Ana L. Reyes-Ramírez, José Pedraza-Chaverri, Omar N. Medina-Campos, Isabel Medina-Vera, Juan G. Reyes-García, Armando R. Tovar, Aurora E. Serralde-Zúñiga

**Affiliations:** 1 Servicio Nutriología Clínica, Instituto Nacional de Ciencias Médicas y Nutrición Salvador Zubirán, Mexico City 14080, Mexico; 2 Sección de Estudios de Posgrado e Investigación, Escuela Superior de Medicina, Instituto Politécnico Nacional, Mexico City 11340, Mexico; 3 Departamento Fisiología de la Nutrición, Instituto Nacional de Ciencias Médicas y Nutrición Salvador Zubirán, Mexico City 14080, Mexico; 4 Unidad de Investigación, Hospital Regional de Alta Especialidad de la Península de Yucatán, Mérida 97130, Mexico; 5 Departamento de Metodología de la Investigación, Instituto Nacional de Pediatría, Mexico City 04530, Mexico; 6 Departamento de Biología, Facultad de Química, Universidad Nacional Autónoma de México, Mexico City 04510, Mexico

**Keywords:** intestinal failure, oxidative stress, n-3 polyunsaturated fatty acids, fish oil, parenteral nutrition

## Abstract

Type II intestinal failure (IF-II) is a condition in which the gastrointestinal tract is compromised. Liver complications may occur because of the pathology and/or prolonged use of parenteral nutrition (PN); oxidative stress has been implicated as one of the causes. Lipid emulsions containing n-3 polyunsaturated fatty acids (PUFAs) have been proposed for the treatment. We aimed to evaluate the effect of 7-day n-3 PUFA supplementation on oxidative stress in IF-II patients receiving PN. This was a randomized, controlled, double-blinded, pilot trial of adult patients with IF-II, receiving either conventional PN (control) or PN enriched with n-3 PUFAs (intervention). Twenty patients were included (14 men, 49 ± 16.9 years), with the ANCOVA analysis the glucose (*p* = 0.003), and direct bilirubin (*p* = 0.001) levels reduced; whereas the high-density lipoprotein cholesterol (HDL-C) increased (*p* = 0.017). In the random-effect linear regression analysis, a reduction (*p* < 0.0001) in the malondialdehyde (MDA) level was found in the intervention group when the covariables age, HDL-C level, and alanine aminotransferase activity were considered. After 1 week of PN supplementation with n-3 PUFAs, the marker levels of some oxidative stress, blood lipids, and hepatic biomarkers improved in patients with IF-II.

## 1. Introduction

Intestinal failure (IF) is the loss of intestinal function, resulting in limited or no nutrient, water, and electrolyte absorption. Therefore, total parenteral nutrition (PN) is required to maintain health and growth in patients with IF [1,2]. Three IF classifications have been proposed, based on functionality (types I, II, and III), pathophysiology (short intestine, intestinal fistula, intestinal dysmotility, mechanical obstruction, and extensive mucosal disease of the small intestine), and clinical condition of chronic IF [1,2,3].

According to functionality classification type I IF is self-limited and resolves quickly. Type II is an acute but prolonged condition, often occurring in metabolically unstable patients, requiring multidisciplinary care and PN for weeks or months. Type III is chronic IF, and only 20–50% of patients achieve PN withdrawal after 2 years [3]. The global incidence of IF is unknown; however, in Europe, the estimated annual incidence of IF-II is 9 cases per million people and that of IF-III is 5–20 cases per million [4]

For the prescription of PN, international guidelines recommend using indirect calorimetry or energy expenditure prediction equations for energy prescription; whereas, macronutrients should be considered based on patient requirements [5]. Lipid emulsions (LEs) are recommended to avoid deficiencies in essential fatty acids (FAs). All lipid emulsions are formulated to be similar as chylomicrons, therefore, once they enter in the blood vessels can be hydrolyzed by lipoprotein lipase into HDL or LDL particles [6,7]. The infusion of 1–2 g/kg lipids per day has been shown to be safe and well tolerated by patients, although prolonged use of LEs can sometimes cause liver damage [3].

The use of PN in patients with IF is indispensable, however it can be associated with mechanical, infectious, and metabolic complications, which may lead to long-term liver complications [3,5,8,9]. One of the complications is IF-associated liver disease (IFALD). Its pathogenesis is related to not only conditions such as excessive caloric intake, bacterial overgrowth, and catheter-related bloodstream infections, but also a lack of prolonged enteral stimulation, alterations in the circulation of bile acids, and changes in the intestinal microbiota [10]. Another important factor related to IFALD is the infusion and type of LEs prescribed in the PN; the infusion of over 1 gram per kilogram of body weight per day and exclusive use of soy oil (SO) LEs has been related to liver damage [11]. Furthermore, it has been described in patients with IF-III, and hospitalized patients with IF-II might also be at a risk of IFALD development [12,13,14].

As in other chronic diseases, inflammatory response mechanisms contribute in the pathogenesis. In the alterations of the bile acids circulation the signaling of farnesoid-X receptor (FXR)/fibroblast growth factor-19 (FGF19) is dysfunctional. A lack of enteral stimulation leads to an increase in intestinal permeability, and therefore, a loss of epithelial barrier function, this can be due to the overproduction of proinflammatory cytokines and changes in the gut microbiota; one of the consequences of the increased intestinal permeability is the translocation of lipopolysaccharide (LPS, known as a pro-inflammatory component), which is produced by bacteria in the microbiota [10,15]. When this translocation occurs, LPS enters the portal circulation, causing the liver to release macrophages, inflammatory cytokines, lipid mediators, and reactive oxygen species (ROS) [15,16]. Watanabe et al. found that chronic inflammation (assessed by C reactive protein average in the years of the disease) is an important risk factor in patients with Crohn’s disease to develop IF with an odds ratio of 1.015 (95% CI 1.002–1.027) [17]. Osowska et al. found that when comparing the three most common LE use in patients with IF-III with PN (SO, olive oil (OO) and a mixture containing fish oil (FO)), inflammation markers as interleukin 8 and C reactive protein were lower in the group of patients which had FO LE [18].

ROS and acetaldehyde induce oxidative metabolism in the liver, which, coupled with the decrease in antioxidant enzymes, such as glutathione, enhances oxidative stress. Reduced glutathione (GSH) has an important relationship with lipoperoxidation, as it can bind to free radicals arising from lipoperoxidation and reduce the amount of hydrogen peroxide formed in cells. The measurement of different markers of liver damage can indicate the level of damage caused. For example, malondialdehyde (MDA) is a marker of damage to cell membranes or lipids, and the quantification of GSH or oxidized glutathione (GSSG) shows damage at the cytoplasmic level [19]. Therefore, oxidative stress involves an imbalance between antioxidants and oxidants in favor of the oxidants, promoting alterations in cells, tissues, and organs. Alterations in the intestinal structure include decreased villus height, increased cellular infiltration, and mucosal sloughing, accompanied by increased aspartate aminotransferase (AST) and alanine aminotransferase (ALT) levels, which are related to hepatic injury [20].

Additionally, LEs are susceptible to lipoperoxidation [21,22]. Owing to the complications associated with the exclusive infusion of SO lipids rich in n-6 polyunsaturated fatty acids (PUFAs) in the PN, new combinations containing not just SO have been developed. LEs derived from FO contain high concentrations of n-3 PUFAs, especially docosahexaenoic acid and eicosapentaenoic acid, both of which have been shown to have anti-inflammatory properties [23,24].

There has been an increasing search for strategies to prevent or reduce oxidative damage in IF. One of the strategies involves the use of intravenous FO LE containing n-3 PUFAs to decrease inflammation, oxidative stress, and apoptosis [25]. When n-3 PUFAs are administered orally they have to be absorbed by enterocytes, however, they are not fully absorbed, nor do they reach the systemic circulation or tissue compartment, therefore there are some losses through feces. To see results of the accumulation of eicosapentaenoic acid (EPA) and docosahexaenoic (DHA) from oral administration of n-3 PUFAs requires weeks or even months while the parenteral administration could show changes within days [26]. The absorption of n-3 fatty acid ethyl esters can also be enhanced through the use of emulsifiers (egg phospholipids and glycerine), which increases the bioavailability of n-3 fatty acid ethyl esters but also in the lymph. This increases the bioavailability n-3 PUFAs by increasing the efficiency of incorporation into chylomicrons and therefore once they are infused in the blood vessels are hydrolyzed by lipoprotein lipase [27].

When n-3 PUFAs are supplemented with LEs, arachidonic acid is competitively surpassed in the eicosanoid synthesis pathway; furthermore alpha-tocopherol is added in FO LE to avoid lipid peroxidation, which could be produced by the number of double bonds in the LE [8]. At the metabolic level, in vitro tests have revealed that LEs derived from FO inhibit the activation of extracellular signal-regulated kinases (ERK-1 and ERK-2), the levels of which increase in the presence of LPS, and also decrease the protein expression of toll-like receptor 4 that binds to LPS, limiting the decrease in the expression of paraoxonase 1 protein, which is related to oxidative stress [23].

Studies have demonstrated the clinical implications of LEs with n-3 PUFAs in comparison to other LEs [18,25]. Most of these studies have been performed in patients receiving PN at home or for prolonged periods [24,28,29]. However, in a systematic review comparing the use of different LEs, the use of n-3 PUFAs in LEs was found to reduce infection rates by 40%, decrease hospital stay duration by 2.14 days, and improve liver function markers such as AST and ALT [30]. The dose of FO LE in PN has been described as 0.1–0.2 g/kg body weight per day. However, some studies have used LEs at concentrations of up to 0.25 g/kg body weight per day, with safe outcomes and improved IFALD conditions [10,31]. In fact, according to the European Society for Clinical Nutrition and Metabolism (ESPEN) guidelines, it is necessary to identify strategies to reduce the n-6/n-3 PUFA ratio for the treatment of IFALD [1].

Considering that the majority of research related to the effect of n-3 PUFA supplementation in PN is in patients with IF-III, we were interested in performing a similar study but in patients with IF-II. Therefore, the primary aim of this study was to evaluate the effect of 7-day n-3 PUFA supplementation on oxidative stress in PN-dependent patients with IF-II. The primary outcome was the change in MDA level. This is because MDA is the final substrate of lipid peroxidation, therefore it is a good biomarker to identify if this was reduced by the effect of n3-PUFAs supplementation. Furthermore, it has been documented that n3 PUFAs decreased MDA levels through changes in the lipid composition in membranes cell or in circulating lipids [32].

As secondary endpoints we choose to evaluate other oxidative stress markers because the effect of n3 PUFAs is related with the changes of FAs in mitochondrial membrane, that have the potential to maintain its activity, through the modulation of reduced glutathione metabolism. Therefore, a secondary aim was to measure oxidative stress markers such as GSH, GSSG, GSH/GSSG ratio, and antioxidant activity (oxygen radical absorbance capacity (ORAC) value) and compare the changes in parameters between patients who received n-3 PUFA supplementation and those who did not receive supplementation. We also assessed the characteristics of nutritional support administered and determine the IF classification according to the pathophysiology and cause. Furthermore, we compared biochemical parameters before and after intervention, and anthropometrical and clinical parameters at the beginning of the intervention and end of follow-up (as length of hospital stay (LOS), mortality, and time with PN and food reintroduction).

## 2. Materials and Methods

### 2.1. Study Design, Ethics Approval, and Interventions

This randomized, controlled, double-blind pilot trial was performed at the Instituto Nacional de Ciencias Médicas y Nutrición Salvador Zubirán in Mexico City from 2018 to 2022. Patients admitted to the hospital following a diagnosis of IF and requiring PN were followed up. Patients who received PN without enteral stimuli for 28 days were invited to participate in the study. All patients in this study received intravenous LE of 80% olive oil (OO) and 20% SO LE from the first day of PN administration to avoid any deficiency of essential FAs [24,33,34], because is the standard treatment in our hospital. Patients were excluded if they had chronic hepatic or renal failure, had cancer or were immunosuppressed, were on another feeding route, were prescribed with n-3 PUFA (Omegaven^®^, Fersenius Kabi, Bad Homburg, Germany) or probiotic supplementation during their hospital stay or were pregnant or lactating. All patients read and signed a consent letter before the intervention. This study was approved by an appropriate ethics committee and registered at clinicaltrials.gov (NCT03869957). 

Patients were randomly assigned (randomization was performed with a 1:1 ratio using random block sizes by a person who was not involved in patient treatment) to either the control or intervention group. Both groups received the same total amount of LE to meet their nutritional requirements. The control group received conventional PN with intravenous LE of 80% OO and 20% SO LE for 7 days. The intervention group received PN with FO supplementation covering a nutritional requirement of approximately 0.1–0.2 g/kg/day and the rest of the prescribed lipids were administered as intravenous LE of 80% OO and 20% SO LE. In 10 g of FO LE, it contains 1.25–2.82 g of EPA, 1.44–3.09 g of DHA, 0.1–0.7 g of linoleic acid, 0.2 g of alpha linolenic acid, 0.1–0.4 g of arachidonic acid, 2.5 g of glycerol, 1.2 g of egg phospholipids. 

The person who was involved in randomization sent the necessary information to the pharmacy where the PN was prepared to notify the allocation for each patient; the PN admixtures had the same appearance, as intravenous LEs had the same color. Therefore, the formulations were indistinguishable; both patients and investigators were blinded throughout the study. All data were recorded in an electronic database in Microsoft Excel software (version 2306164).

### 2.2. Anthropometry

All patients were weighed using a digital scale (SECA 813). Body composition was analyzed using a multifrequency bioelectrical impedance analyzer (InBody S10, Seoul, Republic of Korea). Anthropometric variables were measured on day 0 and at the end of patient follow-up (30 days after day 7 or at discharge).

### 2.3. Evaluation of Biochemical Parameters

Oxidative, liver, and lipid biomarkers were assessed on days 0 and 7 of the intervention. Blood samples were collected and centrifuged at 1200× *g* for 10 min to separate serum and plasma. The samples were stored at −80 °C until analysis. 

#### 2.3.1. Evaluation of Oxidative and Antioxidant Biomarkers

The level of MDA in plasma was measured using a spectrophotometric method [35]. Briefly, 200 mL of serum/plasma was mixed with 650 mL of 10 mM 1-methyl-2-phenylindole and 150 mL of concentrated HCl. The mixture was incubated at 45 °C for 40 min and centrifuged at 3000× *g* for 5 min. Finally, the optical density of the supernatant was measured at 586 nm. A standard curve was constructed using tetramethoxypropane. Data are expressed as micromole (μM). 

Plasma antioxidant capacity was measured using the ORAC assay [36]. Briefly, 25 mL of Trolox standards and diluted serum/plasma (1:400) were mixed with 25 mL of 153 mM 2,2′-Azobis (2-methylpropionamidine) dihydrochloride and 150 mL of 50 nM sodium fluorescein in 96-well black plates. Fluorescence was measured every minute at 37 °C for 90 min at 390 nm excitation and 478 nm emission wavelengths using a Synergy HT Multi-Mode Microplate Reader (BioTek Instruments, Inc., Winooski, VT, USA). ORAC value was calculated using the net area under the decay curves; it is expressed as μM Trolox equivalents. 

Total glutathione (GSH + GSSG) content and GSSG level in the serum/plasma were measured using a method based on the reaction of GSH with 5,5’-dithio-bis (2-nitrobenzoic acid) (DTNB) to produce 5’-thio-2-nitrobenzoic acid (TNB), which is detectable at 412 nm, and oxidized glutathione-TNB adduct (GS-TNB) [37]. Briefly, 0.2 mL of serum/plasma was deproteinized with 0.1 mL of 0.6% sulfosalicylic acid. This mixture was centrifuged at 8000× *g* for 10 min at 4 °C, and the supernatant was used for measurements. For GSH determination, 20 mL of GSH standards and serum/plasma were incubated for 30 s with 60 mL of 1.68 mM DTNB and 60 mL of 3.3 units/mL of glutathione reductase (for the conversion of GSSG to GSH). After adding 60 mL of 0.8 mM NADPH, the absorbance of the samples was measured at 412 nm every minute for 2 min. 

A standard curve was constructed using GSH. The data are expressed as μM GSH. For GSSG measurement, GSH was derivatized with 2-vinyl pyridine as follows: 100 mL of GSSG standards or serum/plasma was incubated for 1 h with 2 mL of 10% 2-vinyl pyridine, which binds to GSH present in the samples. After adding 6 μL of 16.6% triethanolamine, the mixture was incubated for 10 min to inactivate 2-vinyl pyridine and prevent it from binding to GSH that will be produced in the next step, which is the same as that described above for GSH determination. Data are expressed as μM GSSG.

#### 2.3.2. Determination of LPS and Other Biochemical Parameters

LPS concentration was determined using an ELISA kit (SEB526Ge; Cloud-Clone Corp., Houston, TX, USA), according to the manufacturer’s instructions. C-Reactive protein (CRP) and other biochemical parameters such as ALT, AST, total cholesterol, low-density lipoproteins (LDL-C), and high-density lipoproteins (HDL-C) were measured using a semi-automatic analyzer (COBAS C-111), based on colorimetric enzymatic technique, following the manufacturer’s instructions.

### 2.4. Evaluation of Clinical Parameters

Energy expenditure was assessed using indirect calorimetry (QUARK PFT COSMED, COSMED, Rome, Italy). During the measurement, the patients were in a supine position, calm, without distractions, awake, and with spontaneous breathing (in patients with tracheostomy, this test could not be performed). The equipment recorded oxygen consumption and expiration of carbon dioxide through a mask to calculate the energy requirement. Clinical variables such as LOS, composition of PN, pathophysiology, and cause of IF were extracted from each patient’s electronic file. Muscle functionality was assessed using a hydraulic hand dynamometer (JAMAR, Maarn, The Netherlands).

### 2.5. Statistical Analysis

Categorical variables are expressed as frequency and percentage. Continuous variables are expressed as mean with standard deviation (SD) if they had a normal distribution, and as median and interquartile range (IQR) if not. Normality was assessed using Kolmogorov–Smirnov test and Q–Q plots. A baseline analysis was performed between the intervention groups. An independent *t*-test was performed for quantitative variables, and the chi-square test was used for categorical variables. ANCOVA was performed to compare the difference in each variable adjusted by the baseline variable levels between days 0 and 7 in the groups. Only for the primary outcome (MDA level), a random-effects linear regression analysis was performed with a general model; using stepwise regression with backward selection, a reduced model was obtained to identify if adjusting with other covariates resulted in a significant difference. Statistical analyses were performed using SPSS version 21 software and STATA version 14 software; results with *p* < 0.05 were considered statistically significant.

No formal sample size calculation was possible because no directly comparable study has been published. However, power calculation was performed according to a study that fits best, where the mean MDA level for the test and control was estimated to be 66 and 37, respectively, with a SD of 25 [38]. The power to detect a difference at a significance level of 5% increases the correlation between the baseline value and the change to baseline level. Assuming an R² of 0.3, a sample size of *n* = 7 or *n* = 8 per group results in a power of 65.67% or 72.71%, respectively. Considering a possible loss of 20%, 10 patients per group were included.

## 3. Results

**Figure 1:**
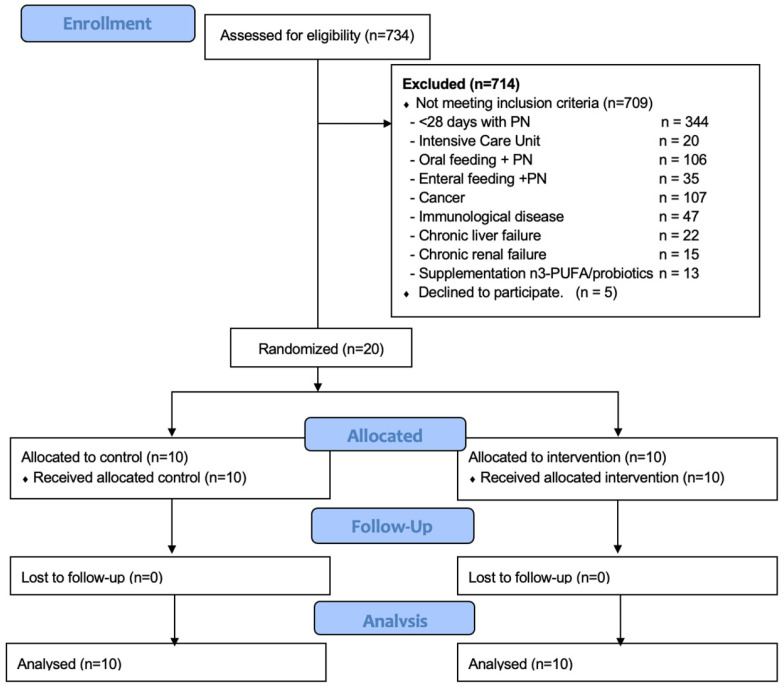
Flow diagram of the patients screened, the motive of exclusion, and those randomized. PN: parenteral nutrition; PUFA: polyunsaturated fatty acids.

We screened 734 individuals, of whom, 20 were randomized to the control (*n* = 10) and intervention (*n* = 10) groups. No patients were lost once they were allocated in each group. Figure 1 shows a flow diagram of the patients screened, the exclusion criteria, and patient randomization.

**Figure 2:**
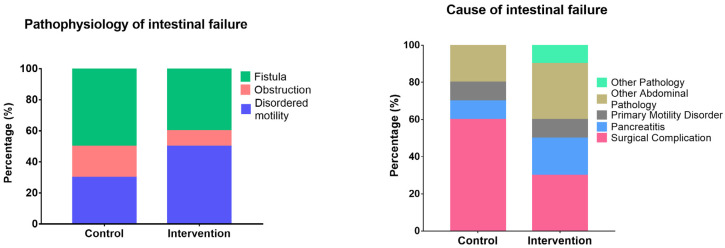
Physiopathology and causes of type II intestinal failure.

Table 1 shows the characteristics of hospital admission of the randomized patients; there was no significant difference between the groups for any of the variables. Figure 2 shows the pathophysiology and cause of IF in the patients, with surgical complications being the leading cause and fistulas being the most common pathophysiology. There were no differences between the groups (*p* = 0.624 and *p* = 0.639, respectively). During the 7 days of intervention none of the patients presented with an active infection which might have altered vital signs or had required the use of antibiotics.

Energy requirements were measured using indirect calorimetry in all but two patients with tracheostomy. PN composition was assessed before the patient started the intervention. There were no significant differences between the groups in energy expenditure or prescription. For patients in the intervention group, FO LE per kilogram of body weight was administered, as shown in Table 2. 

An exploratory descriptive analysis was performed to identify differences between the groups with respect to MDA level using ANCOVA; in the control group, the estimated mean change in MDA level from baseline was 0.219 ± 0.575 nmol/mL compared to −0.148 ± 0.873 nmol/mL in the intervention group. The estimated difference between the groups was −0.470 (95% CI: −1.08 to 0.143) which was not significant (*p* = 0.124). Regarding other oxidative stress markers, the estimated mean change in the GSH level was 0.266 ± 0.667 nM in the control group compared with −0.267 ± 0.622 nM in the intervention group. The estimated difference between the groups was −0.454 (95% CI: −0.915 to 0.007; *p* = 0.053). For the antioxidant activity in the control group the estimated mean change was −62.4 ± 267 μM of Trolox equivalents/mL compared to −66.9 ± 272 μmoles/mL of Trolox equivalents/mL in the intervention group. The estimated difference between the groups was 41.6 mmol of Trolox equivalents/mL (95% CI: −169 to 252; *p* = 0.683) (Table 3).

In addition, we performed random-effects linear regression for MDA level to identify if adjusting with other covariates resulted in a significant difference. Therefore, a general model with the following covariables was used: treatment, age, sex, direct bilirubin (DB), indirect bilirubin (IB), ALT, AST, alkaline phosphatase, CRP, LPS, GSH, GSSG, ORAC, HDL-C, LDL-C, triglycerides, and glucose; a tendency toward significance was obtained (*p* = 0.060). Using stepwise regression with backward selection, we obtained a reduced model that included ALT, HDL-C, age, and treatment, with *p* < 0.0001. The model shows that with higher age (0.029 [95% CI: 0.005–0.053]) and ALT level (0.007 [95% CI:0.002–0.012]), the MDA level increased, and with higher HDL-C levels (−0.043 [95% CI: −0.078 to −0.007]) and n−3 PUFA supplementation [−0.928 (95% CI: −1.67 to −0.189)], the MDA level reduced. With the above results, we proceeded to determine the difference between the models using Hausman test (*p* = 0.986) and verified the reduced model. Given that no difference between models was observed, we retained the reduced model results (Table 4).

The LOS was 61.8 ± 29.2 days for the control group and 78.2 ± 34.6 days for the intervention group (*p* = 0.291). Duration of PN from admission to discharge was also assessed; it was 50.6 ± 26.0 days for the control group and 62.3 ± 34.1 days for the intervention group (*p* = 0.401). The duration from day 0 to the end of the follow-up was 12.0 ± 8.64 days for the control group and 17.0 ± 12.6 days for the intervention group (*p* = 0.334). 

Patients from both groups received PN during the 7 days of intervention; therefore, there was no need to eliminate any patient from the analysis for this reason. Regarding food reintroduction, the control group required a median of 4 days (IQR 1.75–6.5) and the intervention group required 8.5 days (IQR 1.25–20.3) (*p* = 0.689). In the intervention group, two patients completed the follow-up with PN. Furthermore, they were discharged with PN support, changing their diagnosis to chronic IF because the IF could not be resolved. One person died in the control group. However, the cause of death was not related to the underlying condition; during the hospital stay, the patient contracted severe COVID-19, which has a high oxygen requirement, but was denied mechanical ventilation support. No adverse events occurred during the treatment period.

**Figure 3:**
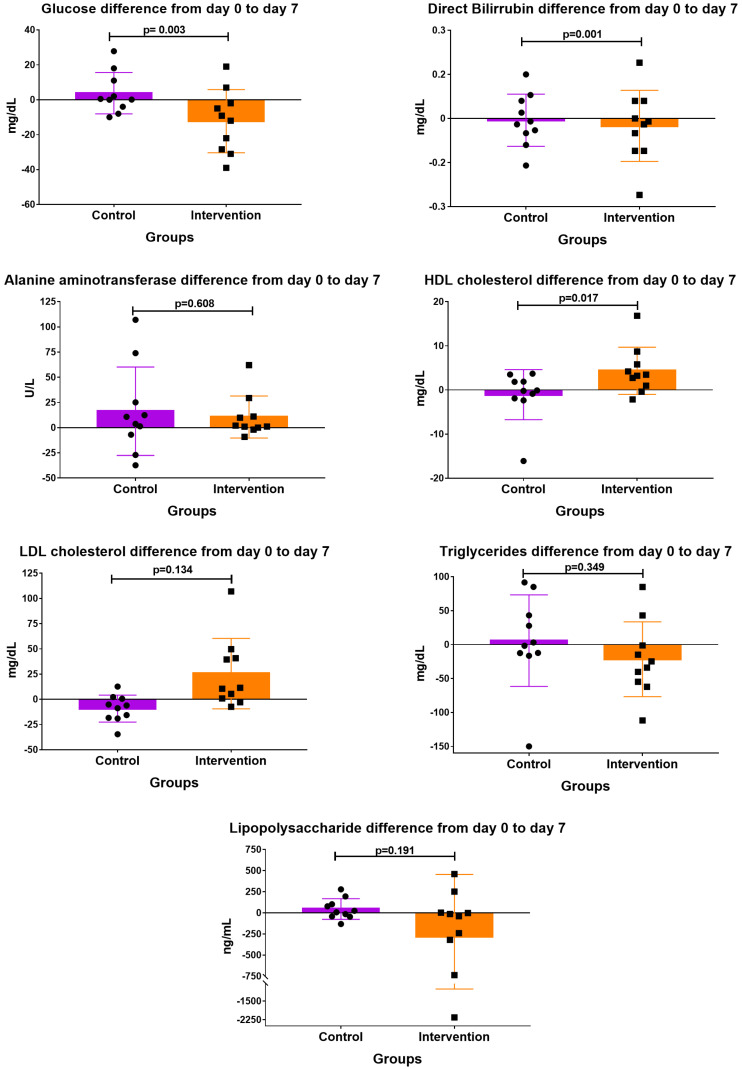
Mean change of biochemical parameters from day 0 to day 7. Data are presented as mean ± SD. Statistical analysis was ANCOVA analysis adjusting for baseline value; *p* value is of the difference of adjusted mean of the pairwise comparison; *p* < 0.05 is considered statistically significant. Each bullet and square on the graphic represent an individual.

Figure 3 shows the results of the other biochemical parameters before (day 0) and after the treatment (day 7). Glucose (*p* = 0.003) and direct bilirubin (*p* = 0.001), showed a significant decrease; HDL-C (*p* = 0.017) significantly increased in the intervention group compared with those in the control group. The LDL/HDL ratio mean change was −0.519 ± 1.04 in the control group compared with 1.63 ± 3.75 in the intervention group. The estimated difference between the groups was 2.00 (95% CI: −0.822 to 4.82; *p* = 0.153). 

Anthropometric measurements could not be obtained in two patients: one due to multiple drains in their body resulting in invalid measurements; the other because of transfer to another hospital at the end of follow-up due to the COVID-19 pandemic. Both patients were a part of the control group. The results are shown in Table 5.

## 4. Discussion

Our results showed that PN enriched with n-3 PUFAs significantly decreased the plasma MDA level when variables such as age, HDL-C, and ALT were considered. This decrease may be partially explained by the fact that n-3 PUFAs can modify the composition of cell membranes [20] and HDL-C composition, making them more resistant to oxidation [39]. Our results showed that n-3 PUFAs supplementation prevented lipoperoxidation but did not increase non-enzymatic and enzymatic antioxidant capacities. Thus, the antioxidant effect on MDA may be a consequence of the infusion of n-3 PUFAs because they have antioxidant properties [40] and can induce the expression of other antioxidant enzymes such as superoxide dismutase, glutathione peroxidase, and heme oxygenase-1 [40,41]. The antioxidant effect of n-3PUFAs has been demonstrated in two ways, through a direct or indirect mechanism. The direct way is through which they neutralize the reactive species and thereby decrease the oxidative damage generated. While the indirect mechanism is through which they modulate the endogenous antioxidant system such as GSH [40]. In addition, the favorable effects in response to oxidative stress are also associated with the property of down-regulating the pro-inflammatory process [42].

The MDA level increases in the plasma of PN-dependent patients with IF compared with that in control individuals [2.3 (1.6–3.4) vs. 1.4 (1.0–1.9) μM] [43] indicating that oxidative stress plays an essential role in this disease, in which inflammation and apoptosis also occur [44]. Other studies, but in patients with IF-III, have shown contrasting results. Kosek et al. compared the effect of the use of FO alone or with OO in the redox state. Compared with a control group (healthy individuals), the MDA level was high in both (FO/-) and (FO/OO) groups; the GSH and GSSG levels showed differences between the groups, but the GSH/GSSG was lower in the (FO/OO) group than in the (FO/-) group [43]. Kosek et al. evaluated patients with long-term use of n-3 PUFAs and non-specific dosages, which may explain the difference in the results between their study and our study (25).

In a cohort study of patients receiving PN at home, Rogulska et al. compared different LEs. Patients administered an admixture of different lipids including SO, medium chain triglycerides (MCTs), OO, and FO (SMOF lipid^®^) had a lower level of MDA than the other group patients. Although there were no significant differences in the ORAC, the SMOF lipid^®^ group had lower values than the other groups [45]. These results are similar to our findings, even though the dosages of n-3 PUFAs were lower than those used in this study.

Regarding the cause and pathophysiology of IF, our results showed that the most common pathophysiology in our patients was fistula, followed by disordered motility and obstructions. Surgical complications, abdominal pathologies, and pancreatitis are the leading causes of IF-II. In a study by Rosas-Flota et al. the major pathophysiology of IF-II fistulas was identified as the primary cause of surgical complications [46]. This could be due to even the research was performed in the same hospital, in the present study the exclusion criteria included pathologies that are common, such as cancer, or a diagnosis of IF-III. In another study performed in IF-II in the United Kingdom, surgical complications (32%) were the most common cause of this type of IF, followed by Crohn’s disease (21%) and vascular ischemia (13%) [47].

Our patients had increased energy requirements; the indirect calorimetry results showed an energy expenditure of over 25 kcal/kg of body weight. Moreover, the measurement was performed without stopping PN infusion, and even then, the respiratory quotient was less than 0.9, which means that mixed substrates were being oxidized due to a catabolic state. In previous studies by Lawinski et al. and Skallerup et al. [48,49], who evaluated the energy expenditure in patients with IF-III, the mean REE was 1181 ± 332 and 1272 ± 245 kcal/day, respectively; our patients showed a mean REE of 1705 ± 371 kcal/day. This difference can be explained by the fact that our patients with IF-II continued to be in an acute but prolonged condition, in which patients continued in a catabolic state. In contrast, the patients included in other studies were in a chronic stable state where the energy requirements did not increase.

Interestingly, some of the biochemical parameters changed within 1 week in favor of the intervention group, such as glucose, direct bilirubin and HDL-C. Similar results were obtained by Antébi et al. in their study on patients who underwent a surgery and required PN for 5 days. Antébi et al. compared SO LE against SMOF lipid^®^, and found that ALT, γ-glutamyltransferase (GGT), and CRP levels were higher in the SO group after 5 days of PN than in the SMOF lipid^®^ group [50]. In the Foil Fact study, involving patients who were admitted to the intensive care unit and required PN, there were no significant changes in biochemical parameters such as liver enzymes between the SO and SMOF lipid^®^ groups, but they found that the triglyceride levels slightly increased in the SMOF lipid^®^ group [51]. In a multicenter study involving patients who received at least 4 weeks of PN, a lower concentration of inflammatory markers, ALT, AST, and total bilirubin, was found in patients administered SMOF lipid^®^ than in patients administered SO LE [52]. Finally, a systematic review showed improvements in liver enzyme markers, such as ALT and AST, when n-3 PUFA LEs were supplemented in PN.

Observing an effect in HDL-C by the consumption of n-3 PUFAs is not new, in a Cochrane study reported an increase in HDL-C when an increase in n-3 PUFAs consumption was present (MD 0.03 mmol/L, 95% CI −0.01 to 0.07, I2 = 66%, in over 14,000 participants) [53]. However, the mechanism is not fully elucidated, different authors have shown parts of it and found different targets where n-3 PUFAs may act to improve HDL-C. The n-3 PUFAs promoted may modify HDL composition and function. It results in increased HDL-C maturation that could be regulated through ApoA-I, which is the principal protein component of HDL-C particles. This process has been regulating the HDL-C maturation through increased ATP Binding Cassette Transporter A1-mediated cholesterol efflux and stimulated enzymatic cholesterol esterification, it is promoting its effects on reverse transport cholesterol [54,55]. Yanai et al. described that the increase in HDL-C due to n-3 PUFAs can be explained by an increased activity of lipoprotein lipase, the reduction of hepatic secretion of apoB-100, and decrease in SREBP-1c (Sterol regulatory element binding protein-1c) [56].

Cartolano et al. found that the consumption of n-3 PUFAs increase large HDL-C subunits while they decrease the small HDL-C subunits, the non-esterified fatty acids in HDL and the activity of CETP (Cholesteryl ester transfer protein) [57]. Additionally, it has been demonstrated that n-3 PUFAs promote macrophage-to-feces reverse transport cholesterol. In human macrophages, in turn, n-3 PUFAs have been shown to significantly increase cholesterol efflux from macrophages [58,59]. Unfortunately, in our study we did not have the opportunity to measure these variables to better elucidate the HDL-C mechanism with our treatment, but we did find a change in this variable unlike a study by Kosek et al. which did not find any difference in this parameter (1.1 mmol/L in FO LE vs. 1.2 mmol/L in FO+ OO LE) which may be explained also because they did not look into the difference of the particles of HDL-C [43].

We did not find differences in other clinical and anthropometric parameters, such as LOS and mortality; we hypothesized that this could be due to the short intervention period or small number of patients; regarding mortality the systematic review reported a non-significant reduction of 16%. However, in terms of LOS, there was a significant reduction of 2.14 days in patients with n-3 PUFA supplementation in PN [30].

Evidence on the effect of PN in IF-II is limited, and previous studies have mainly focused on IF-III or PN administered at home, where the duration of a specific type of PN is generally more than 3 months. Additionally, when analyzing the effect of different LEs, the comparison is mainly between SO LE and LE containing either OO or an admixture that contains various sources of oil, including FO LE. The importance of adding LE containing FO at levels higher than the usually supplemented levels is to reduce the n-6/n-3 PUFA ratio, which has been identified as a key factor to prevent IFALD and oxidative stress in patients with IF [1,60]. To our knowledge, this is the first clinical trial performed to identify if a dosage over 0.1–0.2 g/kg/day of FO LE, used to reduce the n-6/n-3 PUFA ratio, administered for short term (less than 1 week) could change oxidative stress marker levels in hospitalized patients with IF-II.

Our study has some limitations. First, but not intentionally, half of our patients were recruited during the COVID-19 pandemic, which made the follow-up of patients hard and reduced the number of patients admitted to the hospital with pathologies other than COVID-19. Another limitation was that this was a pilot study with a small number of patients, which might have resulted in no significant changes in some of our results and the limitation of measuring other variables as other inflammatory markers such as interleukins that are preferable to be measure with larger samples to avoid misleading results. Additionally, is the short half-life of some of the biomarkers, such as antioxidant activity and GSH, although MDA is a stable and long half-life product of lipid peroxidation. Finally, the amount of alpha tocopherol in the FO LE could be involved in the results of oxidative stress markers changes, which could be an interesting factor to analyze in further studies. The strength of this study is its design, which corresponds to a randomized, double-blind clinical trial.

## 5. Conclusions

In conclusion, n-3 PUFA supplementation within PN resulted in an antioxidant effect in the plasma of patients with IF; this effect was associated with decreased plasma levels of MDA. Our results also support the beneficial role of n-3 PUFAs as potential antioxidants for preventing oxidative stress in IF-II conditions. The results of this study encourage continued research in this category of patients and provide evidence that FO LEs can have a major effect when supplemented with the objective of reducing the n-6/n-3 PUFA ratio, even in a short time, resulting in a good, cost-effective intervention that will need to be investigated in subsequent studies to identify if this therapeutic approach could further be a standard practice specially in those patients who develop cholestasis while using PN.

## Figures and Tables

**Table 1:** Patient’s characteristics at hospital admission.

Variable	Control *n* = 10 Frequency (%)	Intervention *n* = 10 Frequency (%)	*p* Value
Sex			0.329
Male	8 (80)	6 (60)	
Female	2 (20)	4 (40)	
Refeeding syndrome			0.648
Without	1 (10)	2 (20)	
Low	2 (20)	3 (30)	
High	7 (70)	5 (50)	
Surgeries during hospital stay			0.206
0	3 (30)	0 (0)	
1	4 (40)	5 (50)	
2	1 (10)	4 (40)	
Over 3	2 (20)	1 (10)	
Ileocecal valve			0.136
Presence	10 (100)	8 (80)	
Absence	0 (0)	2 (20)	
**Variable**	**Control *n* = 10**	**Intervention *n* = 10**	***p* Value**
Age (years)	45.5 ±19.6	52.5 ± 14.0	0.370
Height (cm) *	172 (163–178)	169 (154–175)	0.481
Weight at admission (kg)	65.5 ± 15.9	72.2 ± 20.2	0.423
Usual weight (kg)	77.1 ± 16.4	76.8 ± 18.4	0.975
Ideal weight (kg)	65.7 ± 7.6	63.7 ± 9.2	0.594
Body mass index	22.8 ± 5.95	26.1 ± 7.18	0.276
Total bilirubin (mg/dL) *	0.525 (0.365–0.710)	0.845 (0.482–1.53)	0.075
Direct bilirubin (mg/dL) *	0.170 (0.142–0.225)	0.320 (0.150–0.582)	0.089
Indirect bilirubin (mg/dL) *	0.360 (0.247–0.480)	0.460 (0.335–0.945)	0.190
Alanine aminotransferase (U/L) *	15.6 (13.0–19.8)	22.8 (10.3–42.3)	0.436
Aspartate aminotransferase (U/L) *	20.1 (14.2–27.3)	23.5 (16.2–35.6)	0.529
Alkaline Phosphatase (U/L)	108.5 ± 69.5	202 ± 109	0.035
Total cholesterol (mg/dL) *	77.5 (54.2–105)	133 (81–237)	0.114
LDL cholesterol (mg/dL) *	45.0 (20.3–67.5)	86.5 (31–152)	0.343
HDL cholesterol (mg/dL) *	16.5 (14.5–20.8)	31.5 (21.5–59.5)	0.057
Triglycerides (mg/dL) *	94.0 (89.0–196)	131 (93.5–159)	0.639
Albumin (g/dL) *	2.61 (2.32–3.29)	2.78 (2.27–3.04)	0.912
Glucose (mg/dL)	116.6 ± 35.3	107.8 ±16.7	0.486
Sodium (mmol/L)	138 ± 5.69	137 ± 4.87	0.834
Potassium (mmol/L) *	4.31 (3.77–4.41)	4.09 (3.92–4.28)	0.393
Chloride (mmol/L)	105 ± 6.12	104 ± 5.21	0.505
Calcium (mg/dL)	8.21 ±0.629	8.07 ± 0.710	0.658
Phosphorus (mg/dL) *	3.35 (2.51–4.17)	3.51 (2.83–3.98)	0.684
Magnesium (mg/dL)	2.02 ± 0.245	1.98 ± 0.215	0.711
C reactive protein (mg/dL) *	13.0 (8.66–17.1)	7.17 (3.73–25.2)	0.739
Days from last surgery to inclusion	29.2 ± 14.9	42.6 ± 26.1	0.248
Days from admission to inclusion	39.2 ± 18.6	49.7 ± 23.6	0.286

Data with normal distribution are presented as mean ± standard deviation, data not normally distributed are presented as the median (25th, 75th percentile) or frequency and percentage. Statistical analysis for categorical variables was performed by Fisher’s exact test or Pearson’s chi-square. Statistical analysis for continue variables was performed with Mann-Whitney U test (*) otherwise with independent *t*-test.

**Table 2:** Nutritional requirements and nutritional support with PN prescribed during the 7 day intervention.

	Control *n* = 10	Intervention *n* = 10	*p* Value
Resting energy expenditure (kcal) ^ Per body weight (kcal/kg)	1728 ± 352 28.5 ± 6.73	1676 ± 416 27.0 ± 5.75	0.773 0.622
Respiratory quotient ^	0.860 ±0.063	0.835 ± 0.065	0.425
Oxygen volume (l) ^	249 ± 50.8	242 ± 57.9	0.805
Carbon dioxide volume (l) ^	214 ±44.3	203 ± 58.7	0.671
Energy from PN Total (kcal/day) Per BW (kcal/kg/day)	1949 ± 275 32.4 ± 7.38	1706 ± 402 29.9 ± 8.29	0.131 0.479
Protein from PN Total (g/day) Per BW (g/kg/day) *	98.5 ± 15.6 1.56 (1.41–1.82)	94.3 ± 21.4 1.44 (1.42–1.91)	0.423 0.853
Dextrose from PN Total (g/day)	282 ± 52.7	243 ± 63.5	0.143
Lipids from PN Per BW (g/kg/day)	0.967 ± 0.207	0.922 ± 0.286	0.695
n-3 PUFAs per BW (g/kg/day)	-	0.183 ±0.078	-
Carbohydrate percentage (%) *	50.3 (49.3–52.3)	48.5 (45.9–51.2)	0.190
Protein percentage (%)	20.4 ± 3.33	21.6 ± 3.50	0.462
Lipid percentage (%)	30.4 ± 4.07	30.3 ± 3.24	0.986

Data with normal distribution are presented as mean ± standard deviation, data not normally distributed are presented as the median. (25th, 75th percentile). PN: total parenteral nutrition; kcal/kg: kilocalories per kilogram of body weight (BW); g/kg: grams of protein per kilogram of BW. Statistical analysis was performed by student’s *t*-test for independent samples, or Mann-Whitney U test (*). ^ two patients were not measured in the intervention group due to the use of tracheostomy.

**Table 3:** Oxidative stress markers. Difference between day 0 and 7.

Variable	Day 0	Day 7	Mean Change (SD)	Difference in Adjusted Mean Change (Test—Control)		
				Mean (SE)	95% CI	*p* Value
MDA (nmol/mL)						
Control (*n* = 10)	2.56 ± 1.43	2.78 ± 1.42	0.219 ± 0.575	−0.470 ± 0.291	−1.08, 0.143	0.124
Intervention (*n* = 10)	2.04 ± 1.17	1.89 ± 1.15	−0.148 ± 0.873			
GSH (nM)						
Control (*n* = 10)	1.82 ± 1.43	2.09 ± 1.41	0.266 ± 0.667	−0.454 ± 0.219	−0.915, 0.007	0.053
Intervention (*n* = 10)	1.86 ± 1.25	1.59 ± 1.01	−0.267 ± 0.622			
GSSG (nM)						
Control (*n* = 10)	0.369 ± 0.229	0.371 ± 0.351	0.001 ± 0.230	−0.003 ± 0.068	−0.147, 0.142	0.970
Intervention (*n* = 10)	0.323 ± 0.265	0.349 ± 0.246	0.026 ± 0.141			
GSH/GSSG ratio						
Control (n = 9)	5.38 ± 7.36	10.9 ± 18.1	5.56 ± 19.0	0.532 ± 5.35	−10.9, 12.0	0.992
Intervention (n = 8)	3.93 ± 4.00	5.26 ± 7.01	1.30 ± 4.71			
ORAC (mmoles of Trolox equivalents/mL)						
Control (*n* = 10)	2024 ± 336	1962 ± 263	−62.4 ± 267	41.6 ± 99.9	−169, 252	0.683
Intervention (*n* = 10)	2112 ± 562	2045 ± 460	−66.9 ± 272			

MDA: malondialdehyde; GSH: reduce glutathione; GSSG: oxidized glutathione; GSH/GSSG ratio: reduce-oxidized glutathione ratio; ORAC: oxygen radical absorbance capacity. ANCOVA analysis was performed adjusting for baseline value. *p* value is of the difference of adjusted mean of the pairwise comparison; *p* < 0.05 is considered statistically significant.

**Table 4:** Stepwise regression with backward selection on the effect of n-3 PUFAs on malondialdehyde levels.

Variable	Coefficient	Standard Error	*p* Value	95% CI
Age	0.029	0.012	0.017	0.005, 0.053
ALT	0.007	0.003	0.008	0.002, 0.012
HDL-C	−0.043	0.018	0.018	−0.078, −0.007
n-3 PUFAs	−0.928	0.377	0.014	−1.67, −0.189

n-3 PUFAs: n-3 polyunsaturated fatty acids. ALT: alanine aminotransferase; HDL-C: high density cholesterol; reduce model of MDA: malondialdehyde. Equation 1.89 + (0.029 × (age)) + (0.007 × (ALT)) − (0.043 × (HDL-C)) − (0.928 × (treatment)).

**Table 5:** Anthropometric measurements. Difference between day 0 and end of follow-up.

Variable	Day 0	End of Follow Up	Mean Change (SD)	Difference in Adjusted Mean Change (Test—Control)		
				Mean (SE)	95% CI	*p* Value
Weight (kg)						
Control (*n* = 10)	62.9 ± 16.6	62.3 ± 15.7	−0.630 ± 3.91	−0.694 ± 1.59	−2.55, 3.94	0.685
Intervention (*n* = 10)	61.4 ± 21.2	60.9 ± 22.0	−0.410 ± 3.07			
BMI (kg/m^2^)						
Control (*n* = 10)	21.4 ± 6.34	21.2 ± 6.04	−0.149 ± 1.29	−0.208 ± 0.625	−1.11, 1.52	0.625
Intervention (*n* = 10)	22.9 ± 8.68	22.8 ± 8.92	−0.120 ± 1.52			
Muscle mass (kg)						
Control (*n* = 8)	24.7 ± 5.78	23.8 ±5.11	−0.063 ± 1.30	−1.71 ± 1.80	−5.55, 2.13	0.358
Intervention (*n* = 10)	24.5 ± 5.37	22.6 ± 6.59	−1.87 ± 4.99			
Fat mass percentage (%)						
Control (*n* = 8)	22.7 ± 18.9	22.9 ± 18.4	0.875 ± 4.64	0.433 ± 1.77	−3.34, 4.21	0.810
Intervention (*n* = 10)	25.2 ± 14.7	26.0 ± 12.1	0.780 ± 5.17			
Visceral fat (cm^2^)						
Control (*n* = 8)	89.1 ± 88.3	89.2 ± 83.7	−5.09 ± 24.1	−1.33 ± 8.12	−18.6, 15.9	0.872
Intervention (*n* = 10)	94.3 ± 58.5	89.7 ± 51.3	−4.59 ± 24.5			
Phase angle (°)						
Control (*n* = 8)	4.53 ± 0.847	4.38 ± 0.888	−0.062 ± 0.244	−0.093 ± 0.219	−0.560, 0.375	0.678
Intervention (*n* = 10)	4.05 ± 1.38	4.02 ± 1.16	−0.030 ± 0.665			
Muscle strength						
Control (*n* = 8)	22.1 ± 12.7	23.6 ± 11.8	1.56 ± 5.72	0.756 ± 1.94	−3.44, 4.95	0.703
Intervention (*n* = 10)	18.4 ± 11.8	17.8 ± 13.1	1.43 ± 2.32			

Normal distribution variables are presented as mean ± standard deviation. Statistical analysis was ANCOVA analysis adjusting for baseline value.

## Data Availability

All relevant data is contained within the article. The original contributions presented in the study are included in the article, further inquiries can be directed to the corresponding author.
